# Supplementary material for: Detection of Anaplasma phagocytophilum in Wild and Farmed Cervids in Poland
Source: Pathogens. 2021 Sep 14;10(9):1190. doi: 10.3390/pathogens10091190 (PMC8471193; doi:10.3390/pathogens10091190)
Supplement: Supplementary file 1 [file pathogens-10-01190-s001.zip › pathogens-1292150-supplementary.pdf]

Table S1. Geographical origin and the number of deer samples.

Species	Origin					
	**Pisz Forest**	**Bolimów Forest**	**Research Station of the Institute of Parasitology**	**Kampinos National Park/ Warsaw urban Forest**	**Stobrawa - Turawa Forest**	**Polesie National Park**
Red deer (n=90)	70	-	15	-	5	-
Fallow deer (n=36)	-	9	27	-	-	-
Roe deer (n=70)	49	5	-	2	14	-
Moose (n=11)	-	-	-	9	-	2

Table S2. 16. *S* rDNA partial gene sequences from *Anaplasma phagocytophilum* aligned with reference sequence derived from GenBank. Nucleotide position numbering is based on the *A. phagocytophilum 16S* rDNA gene sequence, GenBank Accession no. NR_044762.1. In bold sequence changes found in these studies according to reference sequence.

Origin of nucleotide sequence	Accession number	Nucleotide position[2]	
		617	758
**Reference sequence of *16S* rDNA from GenBank**	NR_044762.1	A	T
Red deer	**MZ314416**	**G**	**C**
	MZ317901[1]	A	T
Roe deer	**MZ319389**	A	**C**
	MZ317904[1]	A	T
Moose	MZ317897[1]	A	T

[1]Sequences identical to MZ314415, MZ317900, MZ317903, MZ317898, MZ317899, MZ317902, [2] Nucleotide position determined based the reference sequence.
